# Effects of Unity Prosthetic Elevated Vacuum Suspension System on Minimum Swing Toe Clearance

**DOI:** 10.33137/cpoj.v5i1.36847

**Published:** 2021-10-23

**Authors:** H Gholizadeh, E.D. Lemaire, J Nantel

**Affiliations:** 1 Centre for Rehabilitation Research and Development, Ottawa Hospital Research Institute, Ottawa, Canada.; 2 Department of Medicine, Faculty of Medicine, University of Ottawa, Ottawa, Canada.; 3 School of Human Kinetics, Faculty of Health Sciences, University of Ottawa, Ottawa, Canada.

**Keywords:** Prosthesis, Rehabilitation, Lower Limb Amputation, Gait, Toe Clearance, Transtibial Prosthesis, Mobility, Prosthetic Suspension, Amputation

## Abstract

**BACKGROUND:**

The risk of tripping in people with amputation is greater than that of able-bodied individuals due to reduced toe clearance during the swing phase. Appropriate prosthetic suspension may increase toe clearance by providing more secured attachment between the residual limb and prosthetic socket. Research is lacking on the Unity suspension system's effect on swing toe clearance.

**METHODS:**

Twelve people with transtibial amputation were fitted with the Unity suspension system. After one month accommodation period, the person walked with active (ON) or inactive vacuum (OFF) in a CAREN-Extended virtual reality system, across multiple simulated real-world scenarios. Prosthetics minimum swing toe clearance, and kinematic data, while the vacuum was ON or OFF, were compared with the intact side and a group of 12 able-bodied individuals.

**RESULTS:**

Minimum swing toe clearance (MSTC) and knee flexion angle were larger on the prosthetic side (active and inactive vacuum) compared to both the intact side and the control group. However, hip flexion angle on the prosthetic side was approximately 17% smaller than the control group. Unlike the control group, MSTC with active and inactive vacuum suspension was not significantly different between level walking and other walking conditions. Finally, among all walking conditions, the lowest swing toe clearance for both control and the amputee groups was recorded when the limb was at the top of a side-slope.

**CONCLUSION:**

An effective suspension system could improve toe clearance; however, significant differences were not found between active and inactive vacuum conditions. The likelihood of inappropriate foot contact on side-slope ground might be greater than other walking conditions for both able-bodied and amputee groups, possibly leading to stumbling or falling.

## INTRODUCTION

The vertical distance between the swinging foot's toe region and the ground is defined as minimum swing toe clearance (MSTC) and is a critical gait parameter since it is linked to tripping risk.^1,2^ MSTC in able-bodied individual during level walking is approximately 13 mm, and is sensitive to swing leg ankle, knee, and hip angles.^1,3,4^ People with transtibial amputation have higher risk of tripping and falling than able-bodied individuals, which could be due to prosthetic component malfunction, or poor proprioception. Literature showed any failure in prosthetic suspension system or restricted ankle dorsiflexion may decrease toe clearance in lower limb amputees.^5–7^

Pistoning between the residual limb and prosthetic socket^5,8^ during swing can affect prosthetic length, which could cause insufficient MSTC.^5–7^ Choosing an appropriate prosthetic suspension system to connect the residual limb to the socket is a vital step in the rehabilitation process, leading to improved fit inside the socket and decreased pistoning.^9–10^ Elevated vacuum suspension systems could decrease the pistoning between the residual limb and socket^9–12^ compared to other prosthetic suspension systems, and therefore improve MSTC.

**Figure 1:**
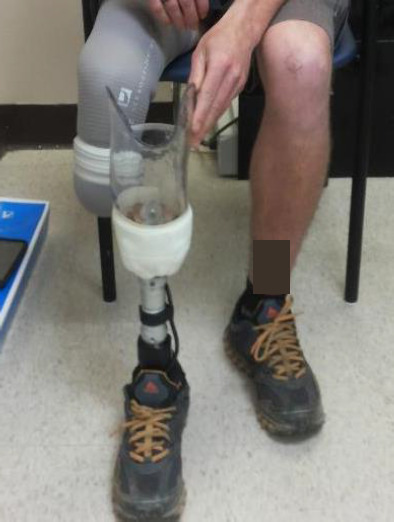
Unity suspension system.

In our previous research, we assessed the effect of Össur's Unity Figure 1 elevated vacuum suspension system (https://assets.ossur.com/library/33281/Unity) on gait parameters while the vacuum was active or inactive.^13–15^ We found significant differences between vacuum conditions for some gait parameters, but differences were small and may not be clinically relevant. However, step length symmetry between intact and prosthetic limbs improved with active elevated vacuum.

The effects of Össur's Unity suspension system on MSTC when walking on community-relevant surfaces with continuous perturbations have not yet been studied and this paper addresses that cap. Walking over non-level surfaces is more challenging than level walking, especially for people with transtibial amputation as they must continually adapt their movement patterns due to the increased biomechanical demands of continuously variable terrain.^13,14,16^ In addition, more challenging walking conditions, may require higher cognitive demand compared to level walking, which could lead to a more cautious gait pattern.^17,18^ Therefore, the purpose of this study was to evaluate the effects of the system on MSTC and kinematics at the hip, knee and ankle during gait across multiple simulated real-world walking scenarios by comparing the prosthetic side, with active and inactive vacuum suspension, to the intact limb. Moreover, MSTC and gait kinematics were compared between the amputee group and 12 able-bodied individuals for the first time in this study. We hypothesized that statistically significant differences will occur between able-bodied participants and the transtibial amputation group regarding MSTC and ankle, knee and hip angles, across all walking conditions. Moreover, Unity users are hypothesized to have larger MSTC on the intact side compared to the prosthetic side with active (ON) or inactive vacuum (OFF). We also hypothesized that positive correlations would occur between MSTC and ankle, knee, and hip angles during different walking conditions. This study can enhance clinicians' and prescribers' understanding of Unity suspension system's effects on MSTC, which can help provide safe mobility for people with limb loss.

## METHODOLOGY

A convenience sample of 12 active people with unilateral transtibial amputation (11 males, 1 female) who used their prosthesis daily was recruited from The Ottawa Hospital Rehabilitation Centre. The participant's mean height was 178.3 (SD=6.4) cm, weight was 90.6 (SD=16.4) kg, age was 57.2 (SD=15.3) years, and time since amputation was 13.1 (SD=20.0) years. Data from a group of 12 able-bodied individuals (11 males, 1 female) from our databases were compared with the amputee group. Control group mean height was 176.6 (SD=7.8) cm; weight was 81.4 (SD=11.0) kg; and age was 38.3 (SD=10.6) years. The Ottawa Hospital Research Ethics Board approved the study protocol and all participants provided written informed consent.

### 2.1. Data collection

A new prosthesis with an Iceross Seal-In V liner and a Pro-flex XC foot with Unity pump was fabricated for each participant and after one month acclimation period, three-dimensional motion analysis was collected in the CAREN Extended virtual reality lab. Participants walked at their own comfortable self-selected walking speed and completed walking trials with vacuum inactive (OFF) or active (ON). The order of active and inactive vacuum was randomized and blinded for the participants. The average self-selected walking speed were 1.07 (SD: 0.23) and 1.03 (SD: 0.20) m/s for inactive and active vacuum respectively. This methodology has been described in detail in our previous publications.^13–15^ The average self-selected walking speed for the control group was slightly higher (mean: 1.29 (SD: 0.06) m/s). Each walking trial was 340 m that included: level walking; down slope (7° decline); up slope (7° incline); right and left slopes (5° slope); medial-lateral translations (platform oscillates in the medial-lateral direction); hilly (platform oscillates in the sagittal plane); and rocky conditions. Right and left slopes were separated into top cross-slope (TS) and bottom cross-slope (BS). During right slope, the right limb was at the bottom of the slope and the left limb was at the top. During left slope, the right limb was at the top and the left limb was at the bottom of the slope.

### 2.2. Data Analysis

Vicon Nexus software version 2.3 (Vicon, Oxford UK) and Visual3D software version 6 (C-Motion, Germantown, MD) were used for gait analysis. Helen Hayes markers set was used in this study. Heel (posterior and lateral side), 5th metatarsal head, and 2nd metatarsal head makers were secured to the shoes. The 5th metatarsal marker was used to track the toe position (toe marker). Minimum toe marker position during swing phase of gait was determined and subtracted from the baseline (toe marker position during the mid-stance) to calculate MSTC. Also, hip, knee, and ankle angles were determined at the point of MSTC. Data were analyzed using Microsoft Excel (version 2010) and SPSS (version 23.0). Shaprio-Wilk tests were used to evaluate data normality and p<0.05 was considered statistically significant. One-way repeated measures ANOVAs were used to find the effect of walking condition on each variable. A one-way ANOVA with a Bonferroni adjustment for multiple comparisons was used to compare between prosthetic (ON, OFF) and intact side and data from 12 able-bodied individuals (control group). Pearson's correlation coefficients (Pearson's R) were used to determine the strength of linear relationship between MSTC and the joint angle at the ankle, knee and hip.

## RESULTS

Mean and standard deviation of MSTC and hip, knee, and ankle angles at the time of MSTC are presented in Table 1. Descriptive statistics showed that MSTC, knee, and hip angle were mostly greater on the prosthetic side (ON and OFF) than on the intact side (Table 1). MSTC and knee angle were greater on the prosthetic side (vacuum ON and OFF) than able-bodied individuals; however, prosthetic hip angle was smaller. Maximum MSTC and knee angle occurred during down slope for both vacuum conditions. However, in the able-bodied group, maximum MSTC occurred during rocky and knee angle occurred during up slope walking. Maximum hip angle occurred during up slope walking for both amputee and able-bodied groups. Lowest MSTC occurred when the limb was at the top of the cross-slope, for all participants. Much larger differences were found between knee and hip angles for amputees than able-bodied individuals. For example, the largest differences between knee and hip angles during down slope walking were approximately 22 degrees for amputees (prosthetic side-vacuum ON) compared to 11 degrees for able-bodied participants.

**Table 1:** Mean and Standard Deviation (in brackets) of minimum swing toe clearance (MSTC, cm) and ankle, knee, and hip angles (degrees) at the time of MSTC. Walking conditions are LW: Level; DS: Down slope; US: Up slope; HL: Hilly; TS: Top cross-slope; BS: Bottom cross-slope; ML: Medio-Lateral; RO: Rocky.

		Walking conditions	INTACT LIMB				PROSTHETIC LIMB			
			MSTC	Ankle	Knee	Hip	MSTC	Ankle	Knee	Hip
12 transtibial amputees	Vacuum ON	LW	1.9 (0.8)	2.2 (2.5)	25.4 (5.5)	16.4 (7.2)	2.2 (0.9)	3.0 (1.6)	31.4 (4.0)	19.1 (6.9)
		DS	2.5 (0.8)	2.8 (2.6)	31.1 (5.9)	14.6 (8.3)	3.1 (0.5)		38.9 (6.6)	17.4 (7.8)
		US	2.1 (0.9)	7.0 (2.8)	34.5 (7.9)	29.5 (9.8)	2.3 (0.9)		35.6 (6.2)	32.9 (9.9)
		HL	1.8 (0.4)	1.9 (1.8)	26.4 (4.6)	17.9 (7.9)	2.6 (0.5)		32.0 (6.6)	20.3 (8.3)
		TS	1.2 (0.9)	4.2 (2.7)	29.4 (5.5)	20.4 (7.6)	1.6 (0.6)		33.3 (7.5)	22.1 (8.0)
		BS	2.4 (0.7)	0.6 (2.4)	24.0 (5.5)	15.0 (7.9)	2.8 (0.6)		30.0 (3.8)	17.8 (7.1)
		ML	1.8 (0.6)	2.2 (2.1)	26.1 (5.1)	17.7 (8.0)	2.2 (0.7)		30.9 (5.0)	20.0 (8.0)
		RO	2.1 (0.6)	1.7 (2.5)	27.5 (5.0)	18.5 (7.8)	2.7 (0.5)		32.1 (4.8)	20.9 (7.6)
		Average (SD)	2.0 (0.4)	2.8 (2.0)	28.1 (3.4)	18.8 (4.7)	2.4 (0.5)		33.0 (2.9)	21.3 (4.9)
	Vacuum OFF	LW	2.1 (1.2)	2.8 (3.2)	25.7 (4.6)	17.3 (7.6)	2.4 (1.1)	3.0 (1.6)	31.7 (6.2)	19.6 (7.1)
		DS	27(1.2)	3.2 (3.3)	30.8 (3.6)	15.2 (8.5)	3.2 (0.7)		38.0 (7.2)	17.5 (8.2)
		US	2.8 (1.3)	7.8 (2.7)	37.6 (6.7)	31.5 (7.9)	2.6 (1.1)		36.7 (7.0)	33.6 (8.5)
		HL	2.4 (1.4)	2.6 (3.7)	27.2 (5.5)	18.7 (8.8)	2.8 (1.4)		32.5 (6.3)	20.4 (8.2)
		TS	1.3 (0.5)	4.6 (3.0)	29.6 (5.6)	20.6 (7.5)	1.8 (0.4)		33.0 (6.5)	23.0 (7.5)
		BS	2.6 (0.6)	1.0 (2.8)	24.6 (4.6)	15.6 (7.1)	3.0 (0.8)		30.8 (6.0)	18.4 (7.0)
		ML	2.2 (1.3)	3.0 (3.1)	26.9 (5.5)	17.9 (7.9)	2.5 (1.1)		31.0 (6.0)	20.3 (7.7)
		RO	2.3 (0.6)	2.9 (3.0)	28.4 (5.5)	18.9 (7.7)	3.0 (0.5)		31.0 (3.8)	20.3 (8.1)
		Average (SD)	2.3 (0.5)	3.5 (2.0)	28.9 (4.1)	19.5 (5.2)	2.7 (0.4)		33.1 (2.8)	21.6 (5.1)
12 able-bodied (average of both legs)		LW	1.6 (0.4)	0.3 (2.0)	25.4 (5.1)	23.1 (5.0)				
		DS	2.4 (0.6)	1.4 (2.7)	29.3 (6.3)	18.7 (6.3)				
		US	2.6 (0.8)	7.3 (3.4)	38.8 (4.5)	38.7 (5.9)				
		HL	2.6 (0.5)	1.1 (2.4)	28.6 (5.4)	25.7 (5.8)				
		TS	1.4 (0.5)	1.6 (1.9)	27.5 (5.7)	26.2 (5.5)				
		BS	2.5 (0.6)	−1.4 (2.2)	24.3 (4.3)	22.7 (4.9)				
		ML	2.2 (0.6)	0.8 (2.3)	27.0 (4.4)	25.1 (5.2)				
		RO	2.9 (0.9)	1.7 (3.0)	28.7 (5.3)	26.6 (6.0)				
		Average (SD)	2.3 (0.5)	1.6 (2.5)	28.7 (4.4)	25.9 (5.8)				

**Figure 2:**
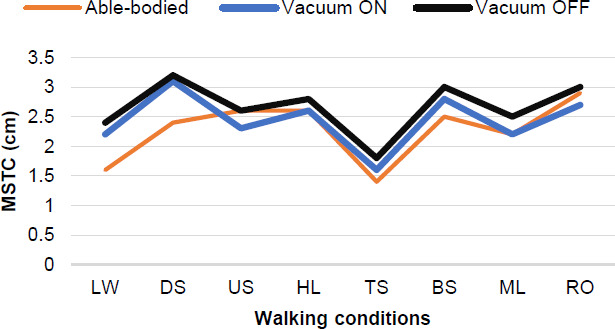
Minimum Swing Toe Clearance in different walking conditions. A comparison between the Unity (vacuum ON and OFF) and Able-bodied group.

One-way repeated measures ANOVA showed no significant MSTC differences between level walking and other walking conditions in the transtibial amputation group (both prosthetic and intact sides) (Table 2, Figure 2). However, in the able-bodied group, MSTC during level walking was significantly smaller than other walking conditions except top cross-slope (Table 2). Hip angle was significantly different (p<0.001) between level walking and up slope walking, for prosthetic and intact sides (vacuum ON and OFF). However, able-bodied group hip angle during level walking was significantly different than other conditions, except bottom cross-slope.

**Table 2:** Mean difference (degrees) and p-value between level walking (baseline) and other conditions. Bold signifies a significant difference. LW: Level; DS: Down slope; US: Up slope; HL: Hilly; TS: Top crossslope; HL: Hilly; TS: Top crossslope; HL: Hilly; TS: Top crossslope; HL: Hilly; TS: Top crossslope; HL: Hilly; TS: Top crossslope; HL: Hilly; TS: Top crossslope; HL: Hilly; TS: Top crossslope; HL: Hilly; TS: Top cross-slope; BS: Bottom cross-slope; ML: Medio-Lateral; RO: Rocky

					INTACT								PROSTHETIC				
			MSTC		Ankle		Knee		Hip		MSTC		Ankle	Knee		Hip	
			Mean Difference	P values	Mean Difference	P values	Mean Difference	P values	Mean Difference	P values	Mean Difference	P values		Mean Difference	P values	Mean Difference	P values
12 transtibial amputees	Vacuum ON	DS	−0.61	1.000	−0.59	1.000	5.72	0.075	1.84	1.000	−0.93	0.063		**7.46**	**0.001**	1.76	1.000
		US	−0.20	1.000	**−4.87**	**0.001**	**9.10**	**0.007**	**−13.08**	**0.000**	−0.11	1.000		4.26	0.159	**−13.77**	**0.000**
		HL	0.11	1.000	0.30	1.000	0.99	1.000	−1.45	0.670	−0.45	1.000		0.65	1.000	−1.19	1.000
		TS	0.69	1.000	**−1.99**	**0.012**	3.96	0.224	**−4.01**	**0.000**	0.53	1.000	-	1.86	1.000	−2.95	0.087
		BS	−0.48	1.000	1.58	0.313	−1.34	1.000	1.44	1.000	−0.65	1.000		−1.36	1.000	1.33	1.000
		ML	0.13	1.000	−0.04	1.000	0.76	1.000	−1.31	0.325	−0.09	1.000		−0.52	1.000	−0.88	1.000
		RO	−0.18	1.000	0.48	1.000	2.15	1.000	−2.11	0.106	−0.58	1.000		0.76	1.000	−1.76	0.590
	Vacuum OFF	DS	−0.64	1.000	−0.37	1.000	**5.09**	**0.021**	2.09	1.000	−0.86	0.110		**6.29**	**0.014**	2.10	1.000
		US	−0.68	0.177	**-4.99**	**0.000**	**11.86**	**0.000**	**-14.23**	**0.000**	−0.23	1.000		**5.04**	**0.015**	**-13.98**	**0.000**
		HL	−0.34	1.000	0.18	1.000	1.53	1.000	− 1.42	1.000	−0.49	0.117		0.84	1.000	−0.75	1.000
		TS	0.78	0.285	**-1.76**	**0.009**	3.88	0.032	**-3.29**	**0.002**	0.55	1.000		1.31	1.000	**-3.36**	**0.003**
		BS	−0.51	1.000	1.84	0.144	−1.11	1.000	1.63	1.000	−0.61	0.995		−0.85	1.000	1.16	1.000
		ML	−0.13	1.000	−0.13	1.000	1.17	1.000	−0.62	1.000	−0.11	1.000		−0.69	1.000	−0.66	1.000
		RO	−0.24	1.000	−0.11	1.000	2.70	0.579	− 1.68	1.000	−0.60	1.000		−0.69	1.000	−0.68	1.000
12 able-bodied (average of both legs)		DS	**-0.74**	**0.003**	−1.10	1.000	3.87	0.222	**4.44**	**0.008**							
		US	**-0.93**	**0.010**	**-7.08**	**0.000**	**13.36**	**0.000**	**-15.56**	**0.000**							
		HL	**-0.92**	**0.001**	−0.88	1.000	3.21	0.226	**-2.56**	**0.043**							
		TS	0.25	0.28	**-1.38**	**0.01**	2.12	0.190	**-3.08**	**0.001**							
		BS	**-0.88**	**0.000**	**1.66**	**0.000**	−1.08	0.670	0.44	0.956							
		ML	**-0.55**	**0.037**	**-0.56**	**1.000**	**1.62**	**1.000**	**-2.00**	**0.021**							
		RO	**-1.21**	**0.003**	−1.49	0.882	3.33	0.245	**-3.56**	**0.023**							

One-way ANOVA results (Table 3) showed significant differences (p<0.05) between the control group and Unity users (vacuum ON and OFF) for MSTC and knee angle during down slope walking (Table 3). Knee angle was significantly different between groups for level and bottom cross-slope conditions. During level walking, MSTC was greater (p=0.041) for Unity users (vacuum OFF) than the control group.

**Table 3:** P values for comparisons between amputee group (prosthetic side) and able-bodied group; A= able-bodied; B= Unity ON; C= Unity OFF. Bold signifies a significant difference. Walking conditions are LW: Level; DS: Down slope; US: Up slope; HL: Hilly; TS: Top cross-slope; BS: Bottom cross-slope; ML: Medio-Lateral; RO: Rocky.

		(A.B)	(A, C)	(B, C)
LW	MSTC	0.316	**0.041**	1.000
	Knee Angle	**0.024**	0.017	1.000
	Hip Angle	0.413	0.571	1.000
DS	MSTC	**0.028**	**0.007**	1.000
	Knee Angle	**0.004**	**0.010**	1.000
	Hip Angle	1.000	1.000	1.000
US	MSTC	1.000	1.000	1.000
	Knee Angle	0.630	1.000	1.000
	Hip Angle	0.296	0.433	1.000
HL	MSTC	1.000	1.000	1.000
	Knee Angle	0.532	0.381	1.000
	Hip Angle	0.274	0.281	1.000
TS	MSTC	0.811	0.148	1.000
	Knee Angle	0.126	0.156	0.515
	Hip Angle	0.498	0.827	1.000
BS	MSTC	0.936	0.312	1.000
	Knee Angle	**0.019**	**0.007**	1.000
	Hip Angle	0.220	0.355	1.000
ML	MSTC	1.000	1.000	1.000
	Knee Angle	0.226	0.202	1.000
	Hip Angle	0.265	0.316	1.000
RO	MSTC	1.000	1.000	1.000
	Knee Angle	0.252	0.746	1.000
	Hip Angle	0.186	0.125	1.000

Pearson correlation coefficients were low between MSTC and ankle, knee, and hip in able-bodied individuals during different walking conditions (Table 4). Correlations were also low between MSTC and hip angle in people with transtibial amputation, on both prosthetic and intact sides (vacuum ON and OFF). Low correlations were found between MSTC and knee angle on the prosthetic side when vacuum was ON. High correlations were found between MSTC and knee angle during hilly and medio-lateral walking when the vacuum was OFF (prosthetic side). A strong correlation was found between MSTC and ankle angle during level walking and down-slope walking on the intact side when vacuum was ON. Moderate and strong correlations were also found between MSTC and ankle angle during different walking conditions on the intact side (vacuum OFF) (Table 4).

**Table 4:** Correlation between MSTC, knee, and hip angles. Correlations greater than 0.5 (moderate and strong correlation) are in bold. Confidence intervals are presented in parentheses. LW: Level; DS: Down slope; US: Up slope; HL: Hilly; TS: Top cross-slope; BS: Bottom cross-slope; ML: Medio-Lateral; RO: Rocky.

			INTACT			PROSTHETIC	
			MSTC-Ankle	MSTC-Knee	MSTC-Hip	MSTC-Knee	MSTC-Hip
12 transtibial amputees	Unity ON	LW	0.65^*^ (0.121, 0.891)	0.15 (-0.464, 0.667)	0.33 (-0.301, 0.760)	−0.01 (-0.581, 0.567)	0.27 (-0.360, 0.731)
		DS	0.69^*^ (0.192, 0.905)	**0.51** (-0.090, 0.838)	0.44 (-0.179, 0.809)	−0.15 (-0.667, 0.464)	−0.12 (-0.649, 0.487)
		US	0.10 (-0.503, 0.637)	**0.54** (-0.049, 0.850)	0.47 (-0.142, 0.822)	−0.23 (-0.710, 0.396)	0.07 (-0.525, 0.619)
		HL	0.48 (-0.130, 0.826)	−0.26 (-0.726, 0.369)	0.07 (-0.525, 0.619)	−0.28 (-0.736, 0.350)	0.25 (-0.378, 0.721)
		TS	−0.31 (-0.750, 0.321)	0.09 (-0.510, 0.631)	−0.30 (-0.746, 0.331)	0.12 (-0.487, 0.649)	0.39 (-0.237, 0.788)
		BS	0.50 (-0.104, 0.834)	0.19 (-0.431, 0.689)	0.27 (-0.360, 0.731)	−0.22 (-0.705, 0.405)	0.05 (-0.539, 0.607)
		ML	0.32 (-0.311, 0.755)	−0.10 (-0.637, 0.503)	0.17 (-0.448, 0.678)	0.25 (-0.378, 0.721)	0.16 (-0.456, 0.672)
		RO	0.42 (-0.203, 0.801)	0.48 (-0.130, 0.826)	0.19 (-0.431, 0.689)	−0.14 (-0.661, 0.472)	0.10 (-0.503, 0.637)
		
	Unity OFF	LW	0.76^*^ (0.330, 0.929)	0.35 (-0.280, 0.769)	0.34 (-0.291, 0.765)	**0.52** (-0.077, 0.842)	0.43 (-0.191, 0.805)
		DS	**0.53** (-0.063, 0.846)	0.39 (-0.237, 0.788)	0.48 (-0.130, 0.826)	0.28 (-0.350, 0.736)	0.34 (-0.291, 0.765)
		US	0.74* (-0.063, 0.846)	0.58* (0.009, 0.866)	−0.02 (-0.587, 0.560)	0.47 (-0.142, 0.822)	−0.05 (-0.607, 0.539)
		HL	0.78* (0.373, 0.935)	0.57* (-0.006, 0.862)	0.36 (-0.270, 0.774)	0.73^*^ (0.269, 0.919)	0.44 (-0.179, 0.809)
		TS	**0.54** (-0.049, 0.850)	0.38 (-0.248, 0.783)	0.14 (-0.472, 0.661)	0.37 (-0.259, 0.779)	0.09 (-0.510, 0.631)
		BS	0.69^*^ (0.192, 0.905)	−0.41 (-0.796, 0.214)	0.12 (-0.487, 0.649)	0.26 (-0.369, 0.726)	0.23 (-0.396, 0.710)
		ML	0.83^*^ (0.489, 0.951)	0.50 (-0.104, 0.834)	0.36 (-0.270, 0.774)	0.62^*^ (0.072, 0.881)	0.25 (-0.378, 0.721)
		RO	**0.56** (-0.020, 0.858)	0.22 (-0.405, 0.705)	0.11 (-0.495, 0.643)	−0.39 (-0.788, 0.237)	0.16 (-0.456, 0.672)
		
12 able-bodied (average of both legs)		LW	0.49 (-0.117, 0.830)	0.24 (-0.387, 0.715)	0.15 (-0.464, 0.667)		
		DS	0.35 (-0.280, 0.769)	0.09 (-0.510, 0.631)	−0.12 (-0.649, 0.487)		
		US	0.45 (-0.167, 0.814)	0.30 (-0.331, 0.746)	0.09 (-0.510, 0.631)		
		HL	0.09 (-0.510, 0.631)	−0.15 (-0.667, 0.464)	−0.03 (-0.594, 0.553)		
		TS	0.35 (-0.280, 0.769)	0.10 (-0.503, 0.637)	−0.01 (-0.581, 0.567)		
		BS	0.40 (-0.226, 0.792)	−0.27 (-0.731, 0.360)	−0.13 (-0.655, 0.480)		
		ML	0.42 (-0.203, 0.801)	0.33 (-0.301, 0.760)	−0.05 (-0.607, 0.539)		
		RO	0.43 (-0.191, 0.805)	0.17 (-0.448, 0.678)	0.35 (-0.280, 0.769)		

## DISCUSSION

In this research, we evaluated the effects of the Unity elevated vacuum suspension system on MSTC during gait. All people in this study had acceptable MSTC, which allowed their feet to clear the ground safely when walking across multiple simulated real-world walking scenarios. Overall, MSTC and knee angles were larger and hip angles were smaller on the prosthetic side (vacuum ON and OFF) than the able-bodied individuals. Different from the control group, no significant MSTC differences were found between level walking and other walking conditions, in the amputee group for both prosthetic and intact sides (vacuum ON and OFF). Unexpectedly, we found no statistically significant differences between vacuum ON and OFF conditions for MSTC, knee, and hip angles.

For the able-bodied group, MSTC was approximately 1.6 cm during level walking, which was similar to the previously reported results.^2,3,19^ Similar to findings by Sinitski et al.,^20^ in amputees, MSTC was greater on the prosthetic side (vacuum ON and OFF) than the intact side and greater than results from able-bodied individuals for most of walking conditions. This outcome differed from the previous literature where the absence of a controllable prosthetic ankle joint caused insufficient MSTC, thereby potentially increasing tripping risk.^6,21,22^ Furthermore, Gates et al., (2012) found that MSTC in people with transtibial amputation was 1.3 times greater on the intact side than the prosthetic side.^17^ Johnson et al., (2014) suggested that using a prosthetic foot with a hydraulic ankle joint could provide adequate toe clearance (2.2 cm) during level walking.^6^ The current study showed that using Unity suspension system could also provide similar MSTC to allow the foot to clear the ground safely during level walking.

This might be due to improved proprioception and socket fit (less pistoning inside the socket) with the Iceross Seal-In V liner.^15^

Literature shows that people adopt a more cautious gait pattern while walking on more challenging walking conditions.^13,14,16^ Moreover, Merryweather et al., (2011) found that MSTC increased significantly when walking on irregular surfaces compared to level walking.^23^ Similarly, we found that able-bodied participants adapted their MSTC to different walking conditions, either by increasing ankle or hip angle. However, there were no significant differences in knee angle between level walking and other conditions, except during upslope walking where knee angle increased approximately 14 degrees (38.8 (SD = 4.5) versus 25.4 (SD = 5.1)) at the point of MSTC. This was expected since incline walking requires the foot to be raised and then contact the ground above the stance limb. People with transtibial amputation also adapted their gait on more challenging walking conditions; however, there were no significant differences in MSTC between level walking and other conditions. The lowest MSTC for amputee and control groups was when the limb was at the top of the cross-slope; therefore, the probability of a stumble scenario on top cross-slopes might be greater than other walking conditions. In the current study, platform tilts to the right or left with a 5° slope; thus, different angles of cross-slope should be tested to determine the risk of tripping in able-bodied and people with transtibial amputation.

Maximum MSTC and knee angle occurred during down slope for the vacuum ON and OFF. Similarly, the literature showed that knee flexion increased during down slope walking since the prosthetic foot (heel) is not able to deform effectively to reach foot-flat.^24^ Amputees in the current study typically had larger MSTC than the able-bodied individuals, therefore MSTC was sufficient to clear the ground and possibly reduce tripping probability. Based on the literature, knee extensors and flexors strength in the amputated side is reduced in comparison with the intact side.^25,26^ Moreover, loss of muscles, tendons, and active ankle dorsiflexion/plantarflexion in the amputated side may compromise function and proprioception.^27^ Therefore, we had expected to have larger MSTC on the intact side compared to the prosthetic side with active (ON) or inactive vacuum (OFF). However, we found larger MSTC in the prosthetic side than the intact side and the able-bodied individuals. This larger MSTC in prosthetic side could be an anticipatory strategy used by people with amputation. Previous research also suggested that, compared to young individuals, older adults actively increase foot clearance as an anticipatory strategy to reduce the risk of contact between the toes and the ground which could cause tripping/falling.^28,29^ Sensinger et al., (2012) also indicated that prosthesis users use different strategies such as vaulting and hip hiking to compensate for inadequate toe clearance.^30^ Larger MSTC in people with transtibial amputation in the current study might also be due to these protective strategies for clearing the ground safely.

Appropriate swing toe clearance could be achieved by reducing effective lower limb length via synchronized ankle, knee, and hip flexion.^20^ One study suggested that increasing hip flexion could increase MSTC.^6^ The current study showed that able-bodied individuals had larger hip angle than the amputee group; nevertheless, MSTC was smaller in the able-bodied group. Moosabhoy and Gard (2006) found that knee and hip have fewer effects on MSTC than ankle.^31^ Similarly, we found no correlation between MSTC and hip angle for amputee and able-bodied groups, whereas higher correlations were found between ankle angle and MSTC in the amputee's intact side.

Prosthetic elevated vacuum suspension system could improve socket fit and proprioception, which could enhance gait symmetry in people with transtibial amputation.^12,32^ Similarly, our previous study showed better proprioception and greater comfort for Unity users with vacuum ON compared to vacuum OFF.^15^ The current study showed non-significant increase of the knee and hip angles in most walking conditions when the vacuum was OFF compared to vacuum ON. Increased knee and hip angles could be an anticipatory strategy used by amputees, possibly due to less proprioception with vacuum OFF, to ensure enough toe clearance. This should be further investigated with a larger number of participants. A high functioning transtibial amputees group with K3 and K4 activity level^33^ participated in the current study. Future research should examine effects of the Unity suspension system on MSTC for lower activity level (K1-K2) to assist in clinical decision-making. One month of accommodation was provided for the Unity suspension system, but no accommodation period was provided for the vacuum OFF condition. Using the prosthesis with vacuum OFF for hours or longer may affect limb volume and socket comfort, where discomfort and inappropriate socket function could affect the gait. Therefore, we only evaluated the immediate effect of vacuum OFF during testing by detaching the distal Unity tube and removing negative pressure inside the socket.

## CONCLUSION

Effective prosthetic suspension system could improve MSTC and might decrease the risk of tripping and falling. The results of this study showed that active people with transtibial amputation could have appropriate MSTC during gait, when using the Unity suspension system. This prosthetic configuration could reduce anticipatory strategies to compensate for the absence of a controllable prosthetic ankle joint.

## DECLARATION OF CONFLICTING INTERESTS

The authors declare that there is no conflict of interest.

## AUTHOR CONTRIBUTION

- **Hossein Gholizadeh:** study design, data collection, data analyses and writing of the manuscript.
- **Edward D. Lemaire:** study design, supervision, review and editing of the manuscript.
- **Julie Nantel:** study design, supervision, review and editing of the manuscript.

## SOURCES OF SUPPORT

This study was financially supported by Mitacs and Össur. All prosthetics components were provided by Össur.

## ETHICAL APPROVAL

The Ottawa Hospital Research Ethics Board approved the study protocol and all participants provided written informed consent.
